# Use of a Large‐Caliber Endoscopic Sheath to Prevent Device Deflection During EUS‐Guided Antegrade Stone Removal

**DOI:** 10.1111/den.70273

**Published:** 2026-09-07

**Authors:** Kenichi Haneda, Akihisa Kato, Michihiro Yoshida

**Affiliations:** ^1^ Department of Gastroenterology and Metabolism Nagoya City University Graduate School of Medical Sciences Nagoya Japan

Endoscopic access to the duodenal papilla can be difficult in patients who have undergone postoperative intestinal reconstruction. EUS‐guided antegrade biliary intervention (EUS‐AI) via a trans‐endosonographically created route (trans‐ESCR) is widely accepted as an alternative method for stone removal [1, 2]; however, the tortuosity of the intrahepatic bile duct often causes device deflection. As a result, force transmission is poor, making it difficult to push stones out of the papilla.

The patient was an 86‐year‐old woman who had undergone distal gastrectomy and Billroth II reconstruction for gastric cancer. She developed common bile duct stone cholangitis (Figure 1a). Transpapillary biliary drainage using a double balloon was attempted, but reaching the papilla was difficult, so EUS‐guided hepaticogastrostomy (EUS‐HGS) was performed (Figure 1b). Subsequently, stone removal was attempted antegrade via the trans‐ESCR. A 0.035‐in. guidewire was guided to the duodenum, and antegrade papillary balloon dilation was performed. The subsequently inserted balloon catheter for stone removal became significantly deflected within the intrahepatic bile duct. Due to this loss of axial force, the catheter was unable to push the stone into the duodenum (Figure 2a, Video 1).

**FIGURE 1:**
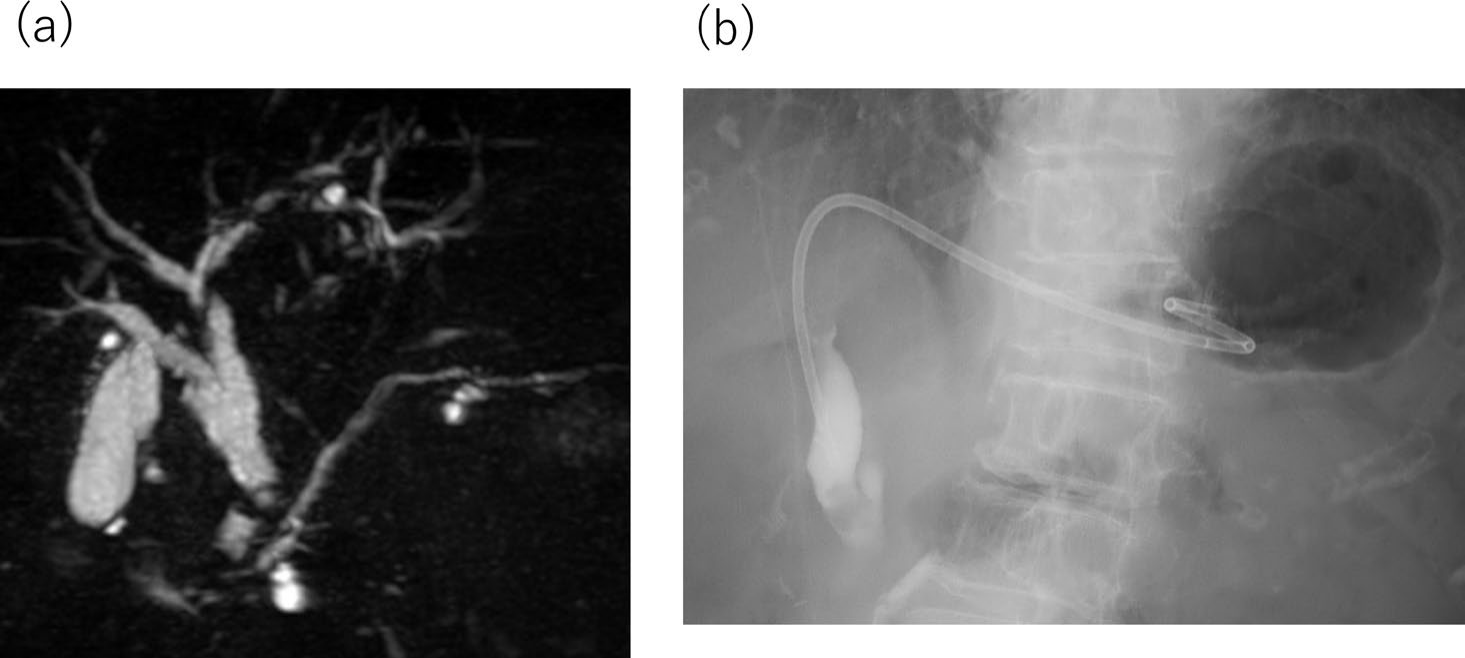
(a) MRCP revealed a signal defect in the distal bile duct that was thought to be a common bile duct stone. (b) Biliary drainage was performed by EUS‐HGS.

**FIGURE 2:**
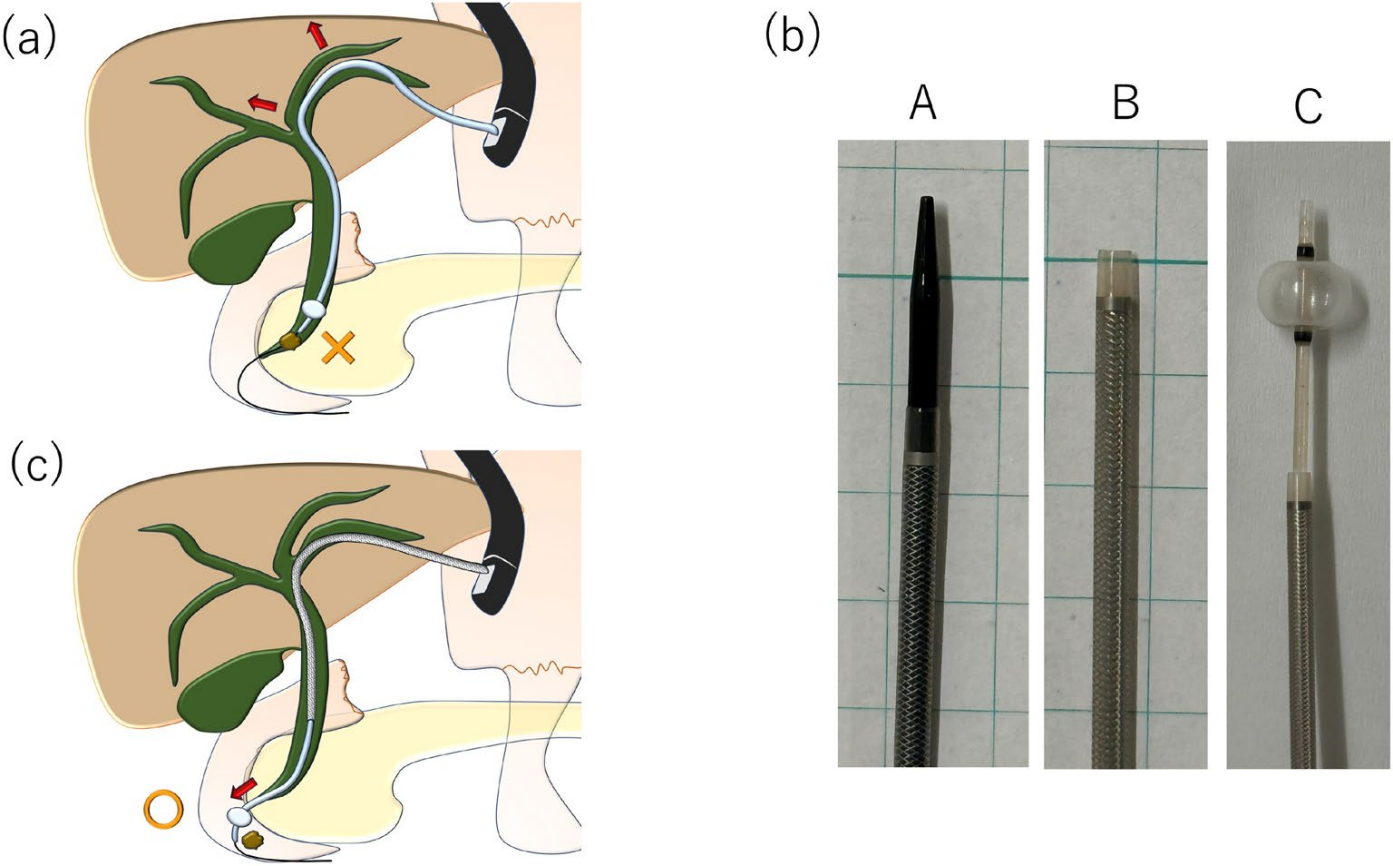
(a) The balloon catheter used for stone removal was deflected within the intrahepatic bile duct, and due to loss of axial force, the stone could not be pushed out into the duodenum. (b) A. Appearance of the modified EndoSheather with an enlarged sheath diameter (inner diameter 8.4 Fr, outer diameter 10 Fr). B. Outer sheath of EndoSheather. C. The 7‐Fr balloon catheter for stone removal in the outer sheath of EndoSheather. (c) The sheath ensured the transmission of force by preventing the catheter from bending, allowing the catheter to pass smoothly through the papilla and remove the stone.

**VIDEO 1:** The large‐caliber endoscopic sheath prevented the deflection of endoscopic devices and facilitated smooth antegrade stone removal during the EUS‐guided procedure. Video content can be viewed at https://onlinelibrary.wiley.com/doi/10.1111/den.70273.

To solve this problem, we used a large‐caliber version of EndoSheather (Piolax Inc., Yokohama, Japan) (Figure 2b) [3, 4, 5]. By advancing the sheath over a guidewire into the bile duct, the sheath served as a stable conduit for the balloon catheter. The sheath effectively prevented device deflection and ensured optimal force transmission, allowing the catheter to smoothly pass through the papilla and successfully remove the stone (Figure 2c).

Therefore, the large‐caliber sheath is highly effective for various trans‐ESCR treatments, including intrahepatic stones. Once a stone is moved into the distal bile duct, the sheath's function of preventing device bending plays an indispensable role in reliable transpapillary removal. This ensures optimal force transmission regardless of the initial stone location.

## Funding

The authors have nothing to report.

## Conflicts of Interest

The authors declare no conflicts of interest.

## Data Availability

The data that support the findings of this study are available on request from the corresponding author. The data are not publicly available due to privacy or ethical restrictions.
